# Draft Genome Sequence of Aeromonas sobria Strain CHT-30, Isolated from a Diseased Rainbow Trout (Oncorhynchus mykiss) in Peru

**DOI:** 10.1128/MRA.00110-20

**Published:** 2020-08-13

**Authors:** Fernando Mesías, Diego Gerpe, Carmen Hurtado, Luis Llanco, Enrique Serrano-Martínez, Jesús L. Romalde

**Affiliations:** a Facultad de Medicina Veterinaria y Zootecnia, Universidad Peruana Cayetano Heredia, Lima, Peru; b Departamento de Microbiología y Parasitología, CIBUS-Facultad de Biología & Institute CRETUS, Universidade de Santiago de Compostela, Santiago de Compostela, Spain; Loyola University Chicago

## Abstract

The Gram-negative bacterium Aeromonas sobria is an opportunistic pathogen that affects humans and animals, including fish. Here, we report the draft genome of strain CHT-30, which was isolated from a diseased rainbow trout in Peru. The genome size is 4.91 Mb, with a G+C content of 57.7%, and the genome includes 4,820 coding sequences.

## ANNOUNCEMENT

Aeromonas sobria is an opportunistic pathogen that affects humans and animals (1, 2). This Gram-negative motile bacterium is present in aquatic environments and produces ulcers and hemorrhages in fish skin (3). However, unlike other fish pathogens of the same genus, such as Aeromonas hydrophila and Aeromonas salmonicida, there is little information on the virulence and antimicrobial resistance factors of A. sobria. A. sobria strain CHT-30 was isolated from the spleen of a diseased rainbow trout (Oncorhynchus mykiss) that was reared in a fish factory in Canta, Peru, and presented hemorrhages and ulcers in the skin. The sample was inoculated in glutamate starch phenol red agar (Sigma-Aldrich, USA) and incubated at 22°C for 24 h. Colonies confirmed as being Aeromonas sobria by PCR (4) and sequencing of the 16S rRNA gene were stored at −80°C in brain heart infusion broth (Liofilchem, Italy) supplemented with 20% glycerol.

A single colony of Aeromonas sobria CHT-30 was inoculated onto trypticase soy agar (Pronadisa, Spain) and incubated at 25°C for 24 h for DNA extraction. The bacterial culture was then resuspended in sterile saline solution (0.85% [wt/vol] NaCl), and the genomic DNA was extracted using the DNeasy blood and tissue kit (Qiagen, Germany), according to the manufacturer’s protocol. For whole-genome sequencing, the purified DNA was sent to Fisabio (Valencia, Spain) for sequencing using an Illumina MiSeq sequencer. The Nextera XT library preparation kit (Illumina, San Diego, CA, USA) was used, following the manufacturer’s instructions, to prepare a genomic DNA library, which was subjected to 2 × 300-bp paired-end sequencing with 50× coverage, producing 1,162,630 reads (∼235-bp read length). *De novo* genome assembly was carried out using SPAdes v. 3.6.12 (5), and the quality of the assembly was evaluated with QUAST v. 5.0.2 (6). The assembly produced 343 contigs (>200 bp) totaling 4,912,126 bp with an average G+C content of 57.67%. The contig *N*_50_ value is 151,159 bp, with the largest contig being 386,878 bp. The resulting draft genome sequence was annotated using the Rapid Annotations using Subsystems Technology (RAST) server v. 2.0 (7), which indicated 4,820 protein-coding sequences. A total of 109 tRNAs were identified with tRNAscan-SE v. 2.0 (8). Default parameters were used for all software.

RAST annotation identified 17 coding sequences related to invasion and intracellular resistance, as well as 16 genes related to iron acquisition and metabolism. Also, the presence of several protein-coding genes linked to resistance to antibiotics and toxic compounds, including those coding for copper homeostasis (10 genes), cobalt-zinc-cadmium resistance (11 genes), copper tolerance (8 genes), a mercury resistance operon (7 genes), arsenic resistance (5 genes), adaptation to d-cysteine (1 gene), resistance to fluoroquinolones (4 genes), multidrug resistance efflux pumps (6 genes), mercuric reductase (2 genes), and resistance to chromium compounds (1 gene), was found. Moreover, RAST analysis revealed 12 phage-coding sequences and 128 genes related to flagellar motility. The genome sequence of Aeromonas sobria strain CHT-30 could contribute to searches for antimicrobial resistance and virulence factors and to comparative genomic studies.

### Data availability.

This whole-genome shotgun sequence has been deposited in DDBJ/EMBL/GenBank under the accession number VEMU00000000. The version described in this paper is the first version, VEMU01000000. The raw reads have been submitted and are available under BioProject number PRJNA546108 and BioSample number SAMN11952138. The raw sequencing reads are available under the accession number SRR11881282.
